# Digestibility and Bioavailability of the Active Components of *Erica australis* L. Aqueous Extracts and Their Therapeutic Potential as Acetylcholinesterase Inhibitors

**DOI:** 10.1155/2015/854373

**Published:** 2015-08-12

**Authors:** Pilar Dias, Pedro L. Falé, Alice Martins, Amélia P. Rauter

**Affiliations:** ^1^Centro de Química e Bioquímica, Departamento de Química e Bioquímica, Faculdade de Ciências, Universidade de Lisboa, Edifício C8, Campo Grande, 1749-016 Lisboa, Portugal; ^2^Institute of Pharmaceutical Science, King's College London, 150 Stamford Street, London SE1 9NH, UK

## Abstract

*Erica australis* L. (Ericaceae) is used in traditional medicine to treat many free-radical related ailments. In the present work, the stability and biological activity of the plant aqueous extracts submitted to an *in vitro* digestive process were investigated. Chemical stability was monitored by HPLC-DAD and LC-MS/MS, while the bioactivities were evaluated through the inhibition of acetylcholinesterase (AChE) and DPPH radical scavenging activity. Both extracts, whose main components were flavonol glycosides, inhibited AChE, showing IC_50_ values of 257.9 ± 6.2 *µ*g/mL and 296.8 ± 8.8 *µ*g/mL for the decoction and for the infusion, respectively. Significant radical scavenging activities were also revealed by both extracts, as denoted by the IC_50_ values for the decoction, 6.7 ± 0.1 *µ*g/mL, and for the infusion, 10.5 ± 0.3 *µ*g/mL. After submission to gastric and pancreatic juices, no remarkable alterations in the composition or in the bioactivities were observed, suggesting that the extracts may pass through the gastrointestinal tract, keeping their composition and therefore their biological properties. Moreover, the bioavailability of the components of both extracts, as studied in a Caco-2 cell model, showed that compounds can permeate the membrane, which is a condition to exert their biological activities. Our results add further support to the potential of *E. australis* for its antioxidant and neuroprotective properties.

## 1. Introduction

*Erica* species (Ericaceae) are used in folk medicine of many countries for their therapeutic properties such as antiviral [1], diuretic [2], anti-inflammatory and antinociceptive [3], antioxidant [4, 5], antiulcer [6, 7], antimicrobial [8], hypolipidaemic [9], analgesic [10], and cytotoxic [11] activities.

The flowers and aerial parts of* Erica australis* L. are used in the Trás-os-Montes region (Portugal) to treat prostate, bladder, and kidneys diseases [12] while, in the Montemuro mountains, the plant infusion is claimed to have anti-inflammatory, diuretic, and sedative properties. Studies involving* E. australis* collected in Algarve were recently reported [13–15]. These authors determined the antioxidant and free-radical scavenging activities of different plant parts of* Erica australis* L. and the phenolic, flavonoid, and amino acid profiles of the leaves and flowers from this plant. In addition, the effects of the plant extracts on Caco-2 cells, fibroblasts, and selected pathogenic bacteria responsible for wound infection were also investigated, showing the potential of this plant as a source of bioactive ingredients that may provide a diversity of health benefits. These properties can be attributed to its chemical constituents, mainly phenolic acids, flavonoids, amino acids, and anthocyanins [14, 16–20].

The present work is focused on* E. australis* collected in the Montemuro region, Portugal, concerning the digestibility and bioavailability of the active components of the plant aqueous extracts and their therapeutic potential as acetylcholinesterase (AChE) inhibitors.

The stability of extracts under gastrointestinal conditions was evaluated by HPLC-DAD-MS/MS before and after the submission to an* in vitro* digestion with artificial gastric and pancreatic juices. Furthermore, the antioxidant and antiacetylcholinesterase activities were monitored throughout the digestive process by the measurement of the DPPH radical scavenger activity and AChE inhibition. The bioavailability of* E. australis* extracts was also evaluated by the permeation of its components through Caco-2 cell monolayers, a model of the intestinal barrier. As to our knowledge, this is the first report on the digestibility and bioavailability of the active compounds from* E. australis* aqueous extracts.

## 2. Materials and Methods

### 2.1. Plant Material

Samples of the flowering aerial parts of* Erica australis* were collected from wild populations in the Tarouca region, Montemuro mountain border. The plant was identified by Professor Ana Isabel Vasconcelos Correia, from the Herbarium of Jardim Botânico, Faculdade de Ciências da Universidade de Lisboa, where a voucher specimen (LISU 236833) is deposited. The infusion was obtained from flowering aerial parts collected by Ervital-Medicinal and Aromatic Plants, Ltd. (Castro Daire, Portugal).

### 2.2. Chemicals

All chemicals were of analytical grade. Acetylcholinesterase (AChE), acetylthiocholine iodide (AChI), 5,5-dithiobis(2-nitrobenzoic acid) (DTNB), and 2,2-diphenyl-1-picrylhydrazyl (DPPH), pepsin, and pancreatin were obtained from Sigma (Barcelona, Spain). DMEM (Dulbecco's Modified Eagle's Medium), HBSS (Hank's Balanced Salt Solution) with and without phenol red, glutamine, Pen-Strep (penicillin and streptomycin mixture), PBS (phosphate buffered saline), and FBS (foetal bovine serum) were bought from Lonza (Verviers, Belgium). HPLC grade water, methanol, and trifluoroacetic acid were obtained from Merck (Darmstadt, Germany).

### 2.3. Extracts Preparation

Plant flowering aerial parts, previously dried at room temperature and away from direct sunlight, were used to prepare the decoction and the infusion (50 g plant/L boiling water). Then, both aqueous extracts were filtered through a Whatman number 1 paper, frozen, and lyophilized in a Heto PowerDry 3000 apparatus.

### 2.4. HPLC-DAD and LC-MS^*n*^ Analysis

The HPLC analysis was carried out in an Elite LaChrom VWR Hitachi Liquid Chromatograph equipped with a Column Oven L-2300 and Diode Array Detector L-2455 (VWR, USA). A column LiChroCART 250-4 LiChrospher 100 RP-8 (5 *µ*m) was used. The extract was analysed by HPLC, injecting 25 *µ*L (1 mg/mL) with an autoinjector and using a gradient composed of solution A (0.05% trifluoroacetic acid) and solution B (methanol) as follows: 0 min, 80% A, 20% B; 20 min 20% A, 80% B; 25 min, 20% A, 80% B. The flow rate was 1 mL/min and the detection was carried out between 200 and 500 nm with a diode array detector.

The LC-MS and LC-MS^*n*^ analyses were carried out on a liquid chromatograph Surveyor Plus Modular LC system connected to a LCQ Duo ion trap mass spectrometer equipped with an electrospray ionization (ESI) source, from Thermo Scientific (Bremen, Germany). The column used was a LiChroCART 250-4 LiChrospher 100 RP-8 (5 *µ*m) column (Merck, Darmstadt, Germany). The extract was analyzed by the injection of the sample (25 *µ*L, 1 mg/mL) using a linear gradient composed of solution A (1.0% formic acid) and solution B (methanol) as follows: 0 min, 70% A, 30% B; 20 min 20% A, 80% B; 25 min, 20% A, 80% B. The mass spectrometer was operated in both positive and negative ion modes in the range* m/z* 120–1000 and the parameters were adjusted in order to optimize the signal-to-noise ratios (S/N) for the ions of interest. Briefly, the nebulizing and auxiliary gas (nitrogen) flow rates were 40 and 20 (arbitrary units) and the capillary temperature was set to 250°C. Collision induced dissociation (CID) experiments were performed by isolating the ions within the ion trap and accelerating them in order to suffer multiple collisions with the background gas present in the ion trap (helium) using a data dependent acquisition mode. The ions of interest were activated by applying a percentage of a supplementary a.c. potential in the range of 0.75–1.75 Vp-p (peak-to-peak) to the end cap electrodes of the ion trap at the resonance frequency of the selected ion (referred to as the Normalized Collision Energy, NCE). The injection times were 50 ms in a full scan and 200 ms in a MS/MS scan. Xcalibur software from Thermo Scientific was used to acquire and process the data.

### 2.5. Antioxidant Activity

Antioxidant activity was measured by the DPPH method, as described in [21] with a slight modification. To a solution of DPPH (1.0 mL, 2% in methanol), 10 *µ*L of plant extract (0–1.0 mg/mL) was added. The mixture was incubated for 30 min at room temperature. The absorbance was measured at 517 nm against a corresponding blank. The antioxidant activity was calculated as

(1) $$\begin{array}{c} AA\left(\;\%\;\right)=100\times \frac{\left(A_{DPPH}-A_{sample}\right)}{A_{DPPH}}, \end{array}$$

where AA is the antioxidant activity, *A*_DPPH_ is the absorption of the DPPH solution against the blank, and *A*_sample_ is the absorption of the sample against the blank. The tests were carried out in triplicate and the extract concentration providing 50% of antioxidant activity (IC_50_) was obtained by plotting the antioxidant activity against the plant extract concentration.

### 2.6. Acetylcholinesterase Inhibition

Acetylcholinesterase enzymatic activity was measured using an adaptation of the method described by Ingkaninan and coworkers [22]. Briefly, Tris buffer (325 *µ*L; 50 mM; pH 8), sample solution (100 *µ*L), and acetylcholinesterase solution (25 *µ*L) containing 0.26 U/mL were mixed in a spectrophotometer cuvette and left to incubate for 15 min at 25°C. Subsequently, solutions of AChI (75 *µ*L, 0.023 mg/mL) and DTNB (475 *µ*L, 3 mM) were added. The absorbance at 405 nm was read during the first 5 min of the reaction and the initial velocity was calculated. A control reaction was carried out using water, which was considered to have 100% activity

(2) $$\begin{array}{c} I\left(\;\%\;\right)=100-\left(\;\frac{V_{sample}}{V_{control}}\;\right)\times 100, \end{array}$$

where *I* is the percent inhibition of acetylcholinesterase, *V*_sample_ is the initial velocity of the extract containing reaction, and *V*_control_ is the initial velocity of the control reaction. Tests were carried out in triplicate and a blank with buffer instead of enzyme solution was used. Results are expressed as the mean ± standard deviation.

### 2.7. *In Vitro* Metabolism by the Gastric and Pancreatic Juices

The assay was adapted from [23]. Gastric or pancreatic juices (1.25 mL) were added to extract solution (1.25 mL, 3 mg/mL) and the mixture was left to incubate at 37°C for 4 h. Samples (400 *µ*L) were taken hourly, added to methanol (400 *µ*L), and centrifuged for 5 min at 5000 ×g. The supernatant was analysed by HPLC and used for the determination of acetylcholinesterase inhibition activity, as well as antioxidant activity, according to the procedures described previously. The gastric juice (100 mL) consisted of pepsin (320 mg), NaCl (200 mg) acidified with HCl to pH 1.2. The pancreatic juice consisted of pancreatin (250 mg) in potassium-phosphate buffer (10 mL; 50 mM; pH 8). All assays were done in triplicate.

### 2.8. Bioavailability Studies by Permeation through Caco-2 Cell Monolayers

Bioavailability studies were performed according to the methodology described previously [24]. For transport and metabolism experiments, the cells were seeded at a density of 2–4 × 10^4^ cells/cm^2^ in 12-well Transwell plate inserts with 10.5 mm diameter and 0.4 *µ*m pore size (BD Falcon). The monolayers were formed after 21–26 days. The integrity of the monolayers was evaluated by measuring the permeability of phenol red and the transepithelial electrical resistance (TEER) with a Millicell ERS-2 Volt-Ohm Meter, from Millipore (Darmstadt, Germany). The membranes were considered fit when the permeability of phenol red from apical to basolateral sides was less than 1% in one hour, or the TEER was higher than 250 Ω·cm^2^. In general, to start the assays, the cells were washed with HBSS and 0.5 mL of the solutions to be tested, in HBSS, was applied into the Transwell inserts (apical side of the cells). The apical solution is composed of 0.5 mg/mL of extract in HBSS. Then, 1.5 mL of HBSS was added to the plate well (basolateral side of the cells).

After 6 hours of incubation at 37°C, 5% CO_2_, the solutions in both sides of the cells were collected and analysed by HPLC. The cells were washed with HBSS and then scrapped and resuspended in HBSS. The cells were sonicated 5 × 10 s and centrifuged 10 min at 5000 ×g, and the supernatant was analysed by HPLC. The concentration of* E. australis* extract used in the bioavailability assays was 1 mg/mL, in HBSS, which was chosen because it did not show toxicity and did not affect the Caco-2 cell membrane integrity.

The percentage of permeation (%) was calculated as the proportion of the original amount that permeated through the monolayer, which was calculated as the amount transported (mol) divided by the initial amount in the apical chamber (mol) [25]. The percentage of compound found inside the cells was also calculated by dividing the amount found in the cell lysate (mol) by the initial amount in the apical chamber (mol). When these amounts could not be calculated in mole by the lack of standard compounds, the proportions were calculated based on the HPLC peak area and volume of solution. For convenience in comparing the values of infusion and decoction, the permeation rate of each compound was also estimated in the equivalent of mol/min using the Lambert-Beer law and was calculated as

(3) $$\begin{array}{c} \text{Permeation Rate}=\frac{\text{Amount}}{\left(\Delta t\cdot A\right)}=\frac{\left(\text{Peak Area}\cdot V\right)}{\left(\Delta t\cdot A\right)}, \end{array}$$

where permeation rate is expressed in mAU·mL, time (*t*) in minutes, peak area in mAU·min, volume (*V*) in mL, and membrane area (*A*) in cm^2^.

### 2.9. Statistical Analysis

The software used was Microsoft Excel 2010 and the results were expressed as means ± standard deviation. Additional analysis of variance (ANOVA) was performed, and a significant difference was assumed at a level of *P* < 0.05.

## 3. Results and Discussion

### 3.1. Composition of* Erica australis* Aqueous Extracts

**Figure 1:**
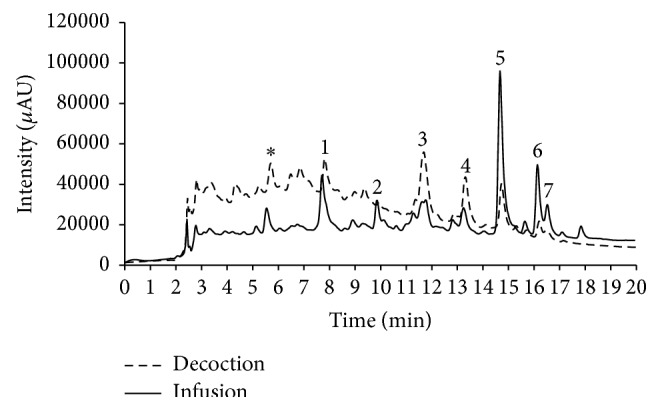
HPLC-DAD chromatogram (200–500 nm) of* Erica australis* aqueous extracts: unknown** (1)**, gossypetin glycoside** (2)**, myricetin 3-*O*-glucoside** (3)**, myricetin 3-*O*-rhamnoside** (4)**, quercetin 3-*O*-rhamnoside** (5)**, kaempferol 3-*O*-rhamnoside** (6)**, and quercetin acetylrhamnoside** (7)**. ^*∗*^ The peak at 5.33 min is the result of an overlap of two smaller peaks. These two minority compounds were in amounts below the detection limit of the MS^*n*^ analysis and therefore could not be identified.

The phytochemical profile of* E. australis* aqueous extracts was evaluated by HPLC-DAD and LC-ESI-MS/MS and chromatograms of both extracts are depicted in Figure 1.

For each peak, the retention times, UV_max_ values, and MS data are summarized in Table 1.

By comparing UV spectra and MS fragmentation patterns with literature data, seven glycosylated flavonols were tentatively identified in both extracts as gossypetin glycoside** (2)** [26], myricetin 3-*O*-glucoside** (3)** [27, 28], myricetin 3-*O*-rhamnoside** (4)** [29], quercetin 3-*O*-rhamnoside** (5)** [30, 31], kaempferol 3-*O*-rhamnoside** (6)** [32], and quercetin acetylrhamnoside** (7)** [33, 34]. However, unambiguous structural elucidation of these compounds will require their isolation from both plant extracts. Compounds** 3** and** 4** were the major constituents of the decoction, while compounds** 5** and** 6** were present in major amounts in the infusion. Some of these glycosides as well as their aglycones (gossypetin, myricetin, kaempferol, and quercetin) were previously identified in other* Erica* species [16, 18–20, 35, 36].

### 3.2. *In Vitro* Antioxidant and Antiacetylcholinesterase Activities

The radical DPPH is widely used to investigate the scavenging activities of several natural compounds or crude mixtures from plants [37].* E. australis* aqueous extracts were proved to have a significant radical scavenging activity as demonstrated by the IC_50_ values for the decoction, 6.7 ± 0.1 *µ*g/mL, and for the infusion, 10.5 ± 0.3 *µ*g/mL. These results are within the range of the IC_50_ values from 296.3 to 4.9 *µ*g/mL found for the leaves, flowers, and branches of* E. australis* evaluated separately, as reported previously by other authors [13].

The inhibition of AChE by both extracts was also evaluated and similar IC_50_ values were obtained for the decoction (257.9 ± 6.2 *µ*g/mL) and for the infusion (296.8 ± 8.8 *µ*g/mL). Extracts of several medicinal plants have been reported to be effective in the inhibition of BuChE and AChE [38], two enzymes involved in Alzheimer's disease. One of the most promising approaches for treating this disease is to enhance the acetylcholine level in the brain using AChE inhibitors [22]. AChE activity is also related to intestinal motility and AChE inhibitors are administered to treat dysphagia, gastric stasis achalasia, abdominal pain, paralytic ileus, vomiting, and constipation [39]. Several plants belonging to the Ericaceae family have shown a very strong activity against AChE and BuChE [38]; however, to our knowledge, no previous studies had been reported for the infusions or decoctions of* Erica* species. The IC_50_ values shown by* E. australis* extracts are lower than the ones found in the literature for many aqueous extracts of plants, such as* Peumus boldus* [40] and* Hypericum* sp. [41], as well as several species used as Portuguese food spices [42], which showed IC_50_ values between 350 and 1, 240 *µ*g/mL. The strong inhibitory activity of* E. australis* aqueous extracts may be attributed to the presence of flavonols, mainly quercetin derivatives, which are reported to inhibit AChE enzyme [43].

### 3.3. *In Vitro* Gastrointestinal Digestion

In order to evaluate if the extracts could reach the intestinal tract without any modification in the chemical composition, the decoction and the infusion were subjected to the action of the gastric and pancreatic juices. Extracts were analysed by HPLC-DAD, and by HPLC-ESI-MS/MS during 4 h and the chromatograms allowed the establishment of the graph shown in Figure 1. No remarkable modifications (data not shown) on the chemical composition of both extracts were observed, which is in accordance with the bioactivity results. There is some controversy about the stability of phenolic compounds submitted to* in vitro* digestions [44]. However, the use of different evaluation methodologies and the composition of food matrix can exert a strong influence on the behaviour of metabolites submitted to* in vitro* and* in vivo* digestive processes.

The antioxidant and anticholinesterase activities of* E. australis* extracts were analysed during the 4 h of the* in vitro* gastric and pancreatic process and results are shown in Table 2.

It can be seen that, after the gastric and pancreatic digestions, the AChE inhibition capacity and the antioxidant activity of the extracts showed small differences that are not statistically significant. The results shown here point out that after a simulated digestive process the activity of the infusion or decoction is not reduced by the gastric or pancreatic juices and can be used for further studies, because it can pass through the gastrointestinal upper tract without any detectable modification. Our results are in accordance with those reported for* Lavandula viridis* extracts [45], whose overall antioxidant and anticholinesterase activities were assured after* in vitro* gastrointestinal digestion.

### 3.4. Bioavailability of the Components

According to the* in vitro* digestive process adopted in this work, the bioactive components of the* E. australis* aqueous extracts are not hydrolysed in the gastrointestinal tract conditions. However, the health improving effects of the aqueous extracts are only effective if the active compounds are able to permeate intestinal barrier to the blood stream. If the compounds can circulate in the blood stream, they may be able to reach the organs where they may be needed as radical scavengers, to decrease inflammation, or to the brain, where they may inhibit acetylcholinesterase activity and can be useful in the symptomatic treatment of Alzheimer's disease.

For each of the main compounds of* E. australis* extracts, the percentage of the initial amount that permeated through the basolateral compartment after 6 hours of incubation with the whole extracts was calculated (Table 3).

The cells were then lysed and the percentage of the compound to be found inside the cells (referred to as intracellular compartment) was calculated. As can be seen in Table 3, all the compounds were able to permeate through the cell membranes to the intracellular and basolateral compartments. Generally, the permeation was similar for the compounds found in the infusion and decoction; however, compounds** 1** and** 5** showed higher percentage of permeation in the decoction, and compounds** 3** and** 4** showed higher percentage of permeation in the infusion. These differences may be due to a higher initial amount of compounds** 1** and** 5** in the infusion and** 3** and** 4** in the decoction, as shown in the HPLC chromatograms (Figure 1), which may cause the saturation of the transporters for these compounds. This fact is also supported by the permeation rate values for these constituents (Table 3), which show very small differences between the infusion and decoction.

Compound** 7** showed a permeation rate significantly higher than all the other compounds in the aqueous extracts. This suggests that its lower polarity may confer it higher lipophilicity, and therefore it may be able to permeate through the membranes independent of transporters. Rodríguez-Roque and coworkers [46] referred to the high bioaccessibility of quercetin and showed that the food matrix exerted a significant influence on the bioaccessibility of different bioactive molecules. An extensive review concerning flavonoid bioavailability and attempts for bioavailability enhancement was recently reported [47], showing that factors like molecular weight, glycosylation, metabolic conversion, and interaction with colonic microflora significantly affect flavonoids' bioavailability.

## 4. Conclusions

The present work evidences that* in vitro* gastrointestinal studies are useful tools to predict the chemical stability, bioactivity, and bioavailability of compounds present in medicinal plant extracts, giving a scientific support to their ethnopharmacological applications. After a simulated digestive process, no significant alterations in the chemical composition or in the antioxidant and anticholinesterase activities of* E. australis* extracts were observed. Moreover, the extracts' components were able to permeate through Caco-2 cell monolayers, denoting their bioavailability. Our work shows scientific evidence to support the potential of this plant as a source of functional ingredients and bioactive molecules for the prevention and treatment of neurodegenerative diseases and other free-radical related ailments.

## Figures and Tables

**Table 1:** Putative identification of compounds present in* Erica australis *aqueous extracts, by HPLC-DAD and HPLC-ESI-MS^*n*^.

Peak	*t*_*R*_ (min.)	*λ*_max⁡_ (nm)	Observed mass [M−H]^−^	*m*/*z* product ions ESI-MS^*n*^ (relative abundance, %)	Compound
**1**	7.95	—	—	—	Unknown

**2**	9.77	248, 275, 331	479	[MS^2^ 479]: 317 (100); [MS^3^ 317]: 299 (100), 287 (19), 271 (47), 255 (12), 231 (11) 195 (24)	Gossypetin-glycoside

**3**	11.65	256, 268, 352	479	[MS^2^ 479]: 317 (48), 316 (100); [MS^3^ 316]: 317 (100), 316 (64), 287 (15), 271 (44)	Myricetin 3-*O*-glucoside

**4**	13.28	258, 271 sh, 351	463	[MS^2^ 463]: 317 (30), 316 (100); [MS^3^ 316]: 317 (100), 316 (99), 287 (19), 271 (68)	Myricetin 3-*O*-rhamnoside

**5**	14.69	256, 269 sh, 348	447	[MS^2^ 447]: 301 (100), 300 (35); [MS^3^ 301]: 301 (100), 271 (34), 255 (23), 179 (62), 151 (35)	Quercetin 3-*O*-rhamnoside

**6**	16.11	269, 348	431	[MS^2^ 431]: 285 (100), 284 (35); [MS^3^ 285]: 285 (100), 257 (16), 255 (9), 229 (7)	Kaempferol 3-*O*-rhamnoside

**7**	16.24	257, 269 sh, 347	489	[MS^2^ 489]: 301 (12), 300 (100); [MS^3^ 300]: 300 (100), 271 (25), 255 (46), 243 (15), 201 (11)	Quercetin-acetylrhamnoside

sh: shoulder.

**Table 2:** Antiacetylcholinesterase and antioxidant activities of *Erica australi*s aqueous extracts before and after *in vitro* gastrointestinal digestion.

Time (h)	AChE (%)				DPPH (%)			
	Infusion		Decoction		Infusion		Decoction	
	Gastric	Pancreatic	Gastric	Pancreatic	Gastric	Pancreatic	Gastric	Pancreatic
0	100.0 ± 27.5	100.0 ± 7.0	100.0 ± 7.6	100.0 ± 5.9	100.0 ± 18.9	100.0 ± 6.9	100.0 ± 5.6	100.0 ± 4.3
1	103.1 ± 16.2	94.0 ± 6.8	98.7 ± 7.8	98.0 ± 3.7	104.5 ± 10.9	101.6 ± 4.3	108.3 ± 3.3	102.4 ± 13.1
2	123.5 ± 18.0	93.2 ± 8.0	97.7 ± 3.7	92.8 ± 1.3	103.3 ± 4.7	98.9 ± 9.7	110.9 ± 8.7	90.2 ± 8.5
3	106.3 ± 16.7	98.2 ± 8.6	104.8 ± 12.8	95.0 ± 10.7	97.6 ± 9.0	100.1 ± 8.6	109.7 ± 17.2	92.7 ± 8.4
4	105.2 ± 10.8	104.1 ± 9.2	104.5 ± 12.7	98.3 ± 10.8	102.6 ± 2.3	100.7 ± 7.9	108.0 ± 3.0	90.9 ± 10.7

**Table 3:** Permeation of compounds through Caco-2 cell monolayers in 6 h incubation period, as percentage of compound found in the basolateral and intracellular compartments and as permeation rate (mAU·mL·cm^−2^).

Compound	Permeation in % of the initial amount				Permeation rate	
	Basolateral compartment		Intracellular compartment		(mAU·mL·cm^−2^)	
	Decoction	Infusion	Decoction	Infusion	Decoction	Infusion
**1**	38.5 ± 6.6	17.2 ± 0.3	21.4 ± 0.4	10.0 ± 0.5	1.68 ± 0.29	2.15 ± 0.03
**2**	46.2 ± 4.5	43.5 ± 4.3	18.8 ± 2.4	15.8 ± 2.1	3.15 ± 0.50	3.49 ± 0.22
**3**	19.2 ± 1.0	37.2 ± 2.2	9.6 ± 0.4	14.4 ± 0.5	1.59 ± 0.13	2.00 ± 0.12
**4**	13.0 ± 2.7	22.9 ± 3.9	5.6 ± 3.1	9.6 ± 0.8	1.82 ± 0.37	2.14 ± 0.38
**5**	10.2 ± 3.5	2.2 ± 1.0	5.7 ± 0.8	2.1 ± 0.2	1.07 ± 0.43	0.81 ± 0.37
**6**	34.5 ± 2.4	31.4 ± 1.1	17.8 ± 0.9	10.1 ± 0.1	2.58 ± 0.30	5.51 ± 0.21
**7**	47.6 ± 0.6	44.5 ± 0.6	31.0 ± 0.6	29.2 ± 0.8	48.70 ± 0.38	48.43 ± 1.07
